# Ancient schwannoma of lumbar spine and review of the literature on paraspinal tumors, the role of preoperative biopsy: a case report

**DOI:** 10.1186/1757-1626-2-9325

**Published:** 2009-12-15

**Authors:** Constantine Antonopoulos, Constantine Lilimpakis, Maria Karagianni, Dimitra Daskalopoulou, Theodoulos Kyriakou, Constantine Vagianos

**Affiliations:** 1 Department of Surgery, Nikea General Hospital, 3D Mantouvalos St, Nikea Pireus, 18454, Greece; 2 Department of Anatomic Pathology, Nikea General Hospital, 3 D Mantouvalos St, Nikea, Pireus, 18454, Greece; 3 Department of Cytopathology, "St. Savas Regional Anticancer, Oncologic Hospital, 171 Alexandras Avenue, Athens, 11522, Greece; 4 Department of Neurosurgery, Nikea General Hospital, 3 D Mantouvalos St, Nikea, Pireus, 18454, Greece

## Abstract

**Introduction:**

Schwannomas are rare encapsulated tumors that derive from the nerve sheath and should be removed due to their infrequent, but existent possibility of malignancy.

**Case presentation:**

We report a case of a mass located in the L5 lumbar spine in a 42 year old man, presented with intermittent lumbar pain. Ultrasound, CT and MRI were used to examine the characteristics of the lesion. Fine needle aspiration showed cytologic characteristics of benign schwannoma and final histological diagnosis was ancient schwannoma. An extraperitoneal approach, through a left paramedian incision was used to approach the site of the mass. The lesion originated from the nerve root of the L4-L5 lumbar spinal space and a complete excision was achieved.

**Conclusion:**

A great variety of tumors should be differentiated when a paraspinal mass is discovered, including neurogenic, neuroendocrine and vascular tumors, as well as malignancies, cystic and inflammatory masses. Fine needle aspiration is a useful and reliable tool in the preoperative evaluation of paraspinal masses. A review of the literature is also presented.

## Introduction

Paraspinal tumors often pose a diagnostic dilemma for the surgeon, due to their commonly silent clinical course and great similarities in radiological characteristics. A variety of heterogeneous lesions should be investigated when a paraspinal lesion is discovered. CT and MRI have improved our ability to differentiate these masses, although significant limitations persist. Preoperative biopsy or fine needle aspiration (FNA) may be very useful, however only the final histological examination can definitively establish the real nature of the lesion.

We present a 42 year old man in whom an ultrasound scan accidentally revealed a paraspinal mass. The mass was surgically removed and the final histology revealed ancient schwannoma of the lumbar spine, a diagnosis that was also suggested by preoperative FNA.

## Case presentation

A 42-year-old man of Greek origin presented with an episode of left intermittent lumbar pain. Abdominal examination did not reveal any tenderness and laboratory data were not remarkable.

**Figure 1:**
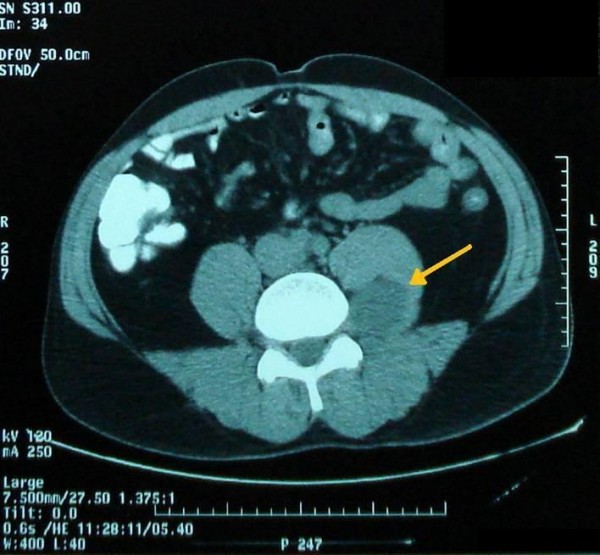
**CT showing a 5 cm mass in the left lumbar paraspinal region, indicated by the arrow**.

**Figure 2:**
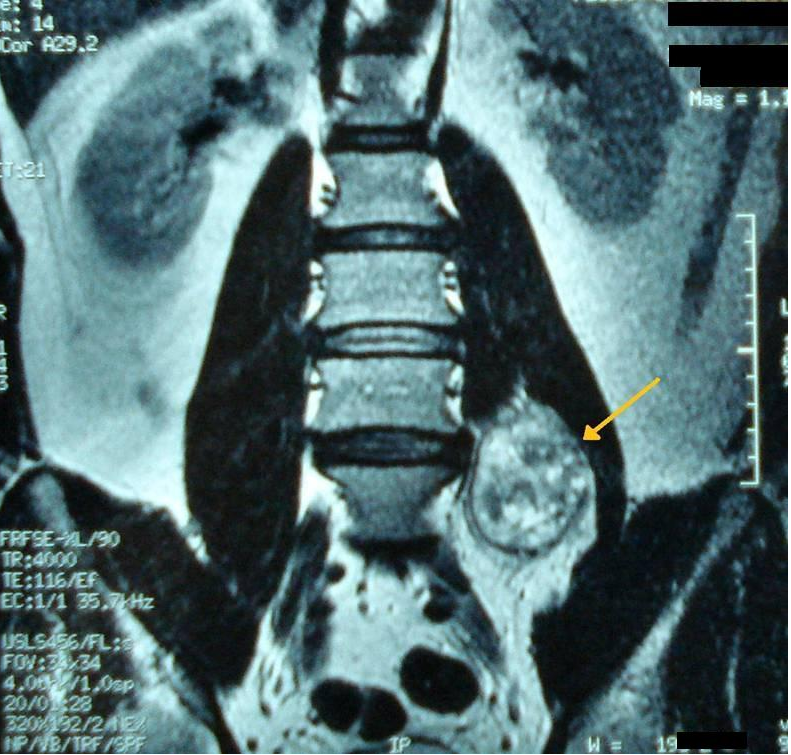
**Abdominal MRI showing a well-defined encapsulated mass, in the left paraspinal region at the L5 level along the posterior side of left psoas muscle**.

**Figure 3:**
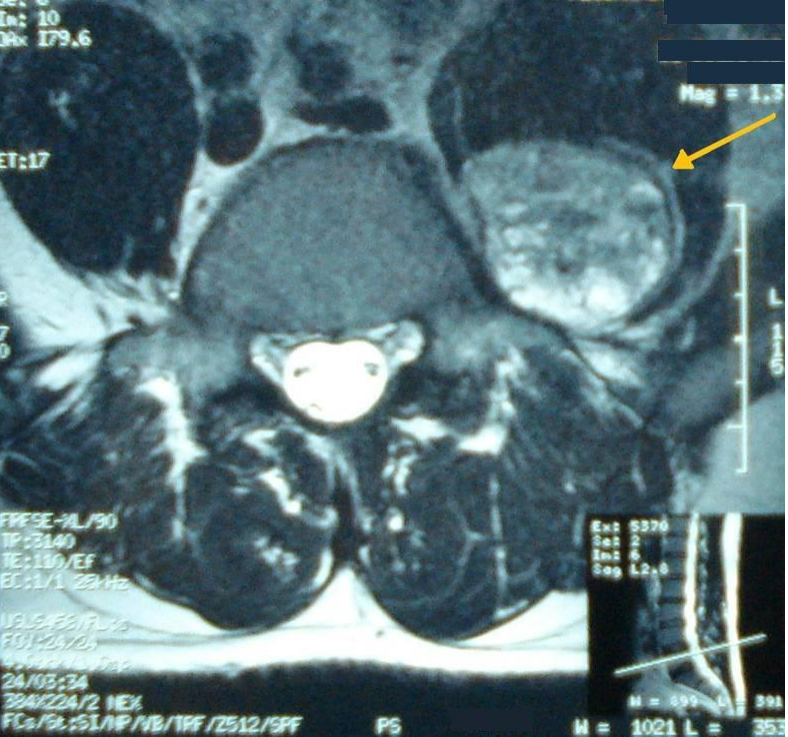
**Lumbar spine MRI showing an encapsulated ovoidal retroperitoneal lesion in the L4-L5 level with scintigraphic uptake and cystic degeneration**.

**Figure 4:**
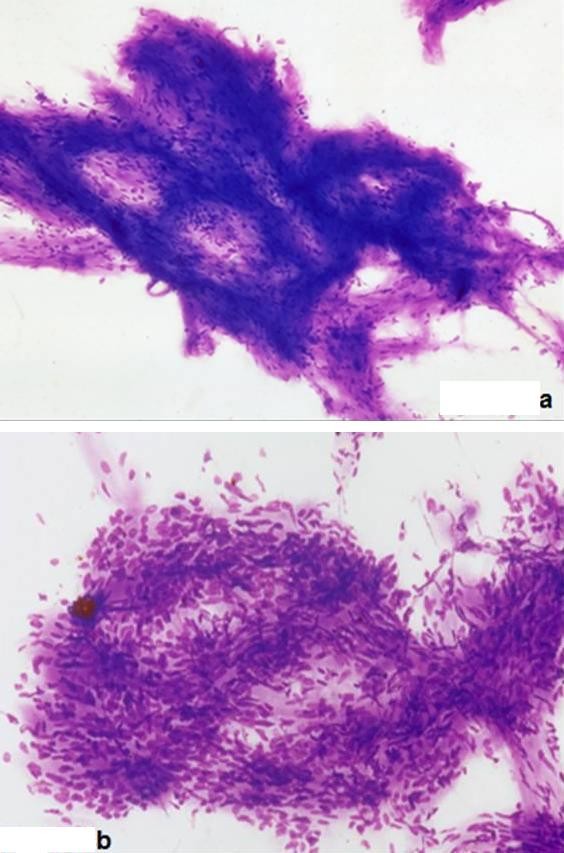
**A, B: Cytologic views of schwannoma**.

Ultrasound sonography revealed a 5 cm, solid, well-defined mass with mixed echogenicity in the left paraspinal region, with no further pathology. A 5 cm, well-defined mass in the left paraspinal region at the L5 level was also showed in CT (Figure 1) and MRI (Figure 2) demonstrating homogeneous low signal intensity on the T1-weighted and a high signal on the T2-weighted abdominal MRI images. There was contrast enhancement, contrary to left psoas muscle that didn't have any scintigraphic uptake. The lesion was in association with the L4-L5 interspinal space and seemed to derive from the spinal root. Lumbar spine MRI (Figure 3) showed an encapsulated ovoidal retroperitoneal lesion in the L4-L5 level along the posterior side of left psoas muscle with great scintigraphic uptake. The tumor demonstrated a cystic degeneration with surrounding collagenous fibrous tissue and was in close relation to L5 spinal root. A CT guided FNA was performed in order to establish a preoperative diagnosis of the tumor. The cytologic examination revealed typical features of benign schwannoma (Figure 4a, b).

The patient was operated with extra peritoneal approach, through a left paramedian incision. The mass seemed to originate from the nerve root of the L4-L5 lumbar spinal space and the excision was complete. On the third postoperative day, the patient complained for strong postural headache that worsened when sitting up and improved after lying down. This was attributed to a leak of the cerebrospinal fluid (CSF) in the spinal membrane, probably caused by minor laceration of the CS canal. The patient's condition was improved with bed rest, paracetamol and hydration. He was discharged on the fifth postoperative day, with no headache, but with a sensory deficit at the site of the left lateral femoral region. The deficit was attributed to left L5 spinal nerve's branch excision, probably occurred during the removal of the mass.

**Figure 5:**
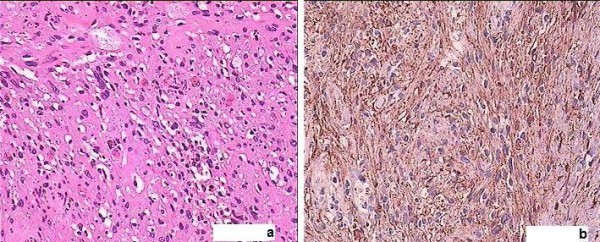
**A: Hematoxylin-eosin stained sections (×40) reveal ancient schwannoma with circumscribed spindle-cell tumor, cellular atypia but no mitotic activity, myxoid degeneration, and vessels with hyalinized walls**. **b**: Strongly positive S100 immunohistochemistry in ancient schwannoma.

Histology showed a well circumscribed spindle-cell tumor with hemorrhage and necrosis, cellular atypia but no mitotic figures, myxoid degeneration, and vessels with hyalinized walls, while S100 immunohistochemistry was strongly positive (Figure 5a, b). Proliferative index Ki-67 was positive, but low. Final diagnosis was ancient schwannoma.

## Discussion

The differential diagnosis of paraspinal lumbar masses includes a variety of lesions (Appendix 1). Schwannoma, neurofibroma, meningioma, ependymoma, sarcoma, ganglioneuroma, tumor arising from lymphoid, connective and bone tissue, abscess, herniated disc, hematoma, spinal arteriovenous malformation (AVM) and spinal aneurysm, as well as metastatic disease should be under consideration [1].

Schwannomas or neurilemmomas comprise neurogenic benign tumors that derive from the nerve sheath. Microscopic evaluation has proved their origin from myelinated Schwann cells, contrary to neurinomas that are nerve-fibre tumors [2]. Schwannomas are rare encapsulated tumors, which are commonly located in peripheral nerves of limbs, head and neck [3]. The first case was described in 1954 and since then 0.7% - 2.7% of all primary schwannomas are located in the retroperitoneum and 0.5% - 1.2% of all retroperitoneal tumors are schwannomas. Retroperitoneal localization affects males and females of mid 50's with a ratio of 2:3. Asymptomatic types are the most common forms, thus making difficult at early stage diagnosis, although nonspecific abdominal or back pain may occur [4]. Motor or sensory deficit, as seen in our patient, entrapment syndrome, and signs due to compression of neighboring structures, including dysuria and constipation are rare clinical manifestations [1,5]. Cellular, glandular, epithelioid, melanotic [1] and ancient types have been described [6]. CT usually shows a non-specific, well defined lesion with low or mixed signal, rarely with areas of cystic necrotic centre [3]. Typical findings of schwannomas in MRI are low signal on T1-weighted and high signal on T2-weighted images [3], while degenerated areas and fibrous tumor capsule are the most useful radiological criteria for the ancient types [4].

Histological stains show two types of tissues; Antoni A and Antoni B, which have been established as the suggestive histological patterns for schwannoma. Nuclear palisading and associated Verocay bodies, which may reflect their prominent extracellular matrix and secretion of laminin are the dominant characteristics of Type A tissue, whereas a loose organization with myxomatous and cystic changes that may represent degenerated Antoni A tissue are the main features of Type B [7]. Sparse mitotic hyperchromatic nuclei and degenerative changes, such as cyst formation, calcification, with only occasional sites of hemorrhage are the major histopathological characteristics of the ancient forms of schwannomas [8].

Although rare, approximately 1% of retroperitoneal schwannomas are malignant [9], especially when combined with von Recklinghausen's disease [10], suggesting complete surgical excision as the best management [2,3,9,11]. Even though surgical excision is the treatment of choice, the surgeon should be aware of spinal nerve involvement, which can cause disabling neurologic deficits.

Apart from schwannomas, a great variety of lesions should be taken into consideration when facing a mass found in lumbar paraspinal region, due to their similarities in radiological imaging and clinical manifestations.

Neurofibromas usually present similar clinical, radiological and histological characteristics, although useful differences may occur. Spontaneous pain and neurological deficit appear more frequently in neurofibromas than in schwannomas and they are more often associated with neurofibromatosis type 1. MRI may reveal multinodular and fusiform shaped masses, especially in the plexiform type of the tumor. Non-plexiform types and schwannomas are of very similar appearance on CT and MRI, a fact which makes their differential diagnosis difficult, although neurofibromas tend to have a more nodular fusiform shape. As regards histological features, presence of axons on axonal staining, is a distinct characteristic of neurofibromas [1].

Extradural meningiomas are difficult to diagnose and can be easily confused with malignant neoplasms [12]. Low signal on T2-weighted images, along with thickening and enhancement of the spinal dura are the most helpful characteristic MRI findings [13]. Intra-operative biopsy is suggested in many studies [14-16].

Ependymoma is another lesion that mimics schwannoma and neurofibroma and poses a diagnostic challenge to the surgeon. Due to the fact that radiological imaging is usually difficult to distinguish among these entities and prognosis depends on the extent of resection, ependymomas should be treated surgically [17].

Ganglioneuromas are slow growing benign tumors that derive from the sympathetic chain [18]. They are rarely located in the retroperitoneal lumbar region and they are often developed during childhood. As these tumors often originate from nerve roots, nerve stimulators and somatosensory evoked potential monitoring are proposed in an effort to minimize postoperative neurological deficits [18].

Paragangliomas are neuroepithelial tumors, which, when located in the lumbar region, may be misdiagnosed as schwannomas or ependymomas. They are usually hypo - isointense to the conus medullaris on T1-weighted and hyperintense on T2-weighted images. Additionally, the presence of hemorrhage and cyst formation in schwannomas and calcifications in meningiomas are useful differential-diagnostic criteria [19].

Extradural lumbar spinal arteriovenous malformations (AVM's) are rare masses that are often associated with vertebral body (cavernous) hemangiomas. Spinal angiography is of the utmost importance and embolization of supply vessels combined, when required, with laminectomy is the treatment of choice [20].

Furthermore, lumbar region can be the location for 20% of spinal metastases, mainly spread from lung, breast, and prostate malignancies. Surgical treatment, assisted by radiotherapy, vertebral body augmentation and spinal radiosurgery can prove beneficial. Ewing sarcomas in children, soft tissue sarcomas and osteosarcomas and chondrosarcomas usually cause bone erosion in CT and MRI images and, therefore, may be preoperatively distinguished [18].

The use of fine needle aspiration (FNA) in above lesions is supported by many studies and along with CT and MRI, it may increase the accuracy of preoperative diagnosis [9]. Tumors like Ewing's sarcoma, osteogenic sarcoma, and neurofibrosarcoma require preoperative chemotherapy and radiation and pelvic desmoids may be reduced in size when preoperative radiation is used, thus making FNA a useful diagnostic tool. Moreover, accuracy, sensitivity and specificity of FNA in mesenchymal tumors have been reported at approximately 90%, showing that FNA may be used with precision for preoperative evaluation [21], although controversy has arisen over the role of FNA in purely cystic lesions.

## Conclusion

It is evident that paraspinal lumbar region can be the source of heterogeneous lesions that extend from the most benign to the most aggressive tumor. It is therefore a necessity for the surgeon to fully understand their pathology, in order to be very comfortable when selecting the optimal treatment. The role of preoperative biopsy is highlighted in several studies and is thought to be essential so as to orientate therapy, determine the operative strategy and avoid overtreatment and major complication.

## Consent

Written informed consent was obtained from the patient for publication of this case report and accompanying images. A copy of the written consent is available for review from the journal's Editor-in-Chief.

## Competing interests

The authors declare that they have no competing interests.

## Authors' contributions

AC undertook a literature review, designed the study and wrote the manuscript. LC undertook a literature review and contributed to the manuscript preparation. KM performed the histological examination. DD performed the FNA examination and contributed to the manuscript revisions. KT contributed to the final version of the manuscript. VC supervised the preparation of the manuscript and reviewed the drafts. All authors read and approved the final manuscript.

## Appendix 1 - Lumbar Paraspinal Lesions

Lumbar Paraspinal Lesions Neurogenic

Schwannomas/Neurinomas/Neurofibromas

Meningiomas

Ependymomas

Ganglioneuromas

Neuroendocrine

Paragangliomas

Vascular

Spinal Arteriovenous Malformations

Spinal Aneurysm

Malignancies

Metastases

Soft tissue sarcomas

Osteosarcomas

Chondrosarcomas

Other

Cystic lesions

Abscess

Haematoma

Herniated disk
